# Medical Department of the University of Buffalo—Commencement

**Published:** 1848-06

**Authors:** 


*Medical Department of the University of Buffalo—Commencement.*—The second annual *commencement* of this institution was on the 14th ult.—Degrees were conferred upon thirty two graduates. In the absence of the Chancellor of the University, Hon. Millard Fillmore, the degrees were conferred by Thomas M. Foote, M. D. The honorary degree of M. D, was conferred on Gustavus A. Rogers, of Bath, Steuben county, N. Y., Jeremiah L. Knight, and Jenks S. Sprague.

The charge to the graduates was delivered by Dr. Flint. The exercises were held at the Washington Street Baptist Church. Music by the choir of the church. Prayers by the Rev. Dr. Tucker. Rev. Chas. Rich, and Rev. M. Schuyler. The public attendance on the occasion, as usual, spoke well for the general interest felt by our citizens in the prosperity of the Institution.

